# Isolated oculomotor nerve palsy due to mesencephalic infarction diagnosed by ZOOM DWI

**DOI:** 10.1186/s12883-025-04021-x

**Published:** 2025-02-07

**Authors:** Shangpei Wang, Yajie Cai, Xiaosan Wu, Sunhong Yan, Longsheng Wang

**Affiliations:** 1 Department of Radiology, the Second Affiliated Hospital of Anhui Medical University, Furong Road NO.678, Hefei, 230601 China; 2 Medical Imaging Research Center, Anhui Medical University, Hefei, 230601 China; 3 Department of Neurology, the Second Affiliated Hospital of Anhui Medical University, Furong Road NO.678, Hefei, 230601 China

**Keywords:** Oculomotor nerve palsy, Mesencephalic infarction, ZOOM DWI

## Abstract

**Background:**

Oculomotor nerve palsy is a common neurological presentation in daily practice.

**Case presentation:**

A 55-year-old man presented with a 3-h history of diplopia and drooping of his bilateral especially left eyelids. Examination revealed an isolated oculomotor nerve palsy consisting of left medial rectus, inferior oblique, superior rectus, inferior rectus with intact pupillary reflexes and bilateral especially left superior palpebral levator. Conventional diffusion weighted imaging (DWI) of the brain showed a suspicious restriction in the left midbrain periaqueductal region. If the clinical symptomatology indicates a lesion in the midbrain, of which a high signal intensity was encountered from neurologically healthy older adults, the limited spatial resolution of conventional axial DWI is an enormous disadvantage. Zonally magnified oblique multislice (ZOOM) DWI correlated with apparent diffusion coefficient map providing higher accuracy for accurate diagnosis can identify signal alterations of mesencephalic interpeduncle area.

**Conclusions:**

This is a rare presentation of isolated oculomotor nerve palsy due to pure mesencephalic infarction especially verified by ZOOM DWI.

## Introduction

Oculomotor paresis is a common neurological presentation in daily practice. There are various causes to oculomotor nerve palsy, with major causes being diabetes, cerebral aneurysms and mononeuritis [1]. Typical mesencephalic infarction mimicking the oculomotor mononeuropathy has been reported only rarely and easy to be ignored by clinicians [2]. Here, we presented a case of pure mesencephalic infarction triggering isolated oculomotor paresis diagnosed by using zonally magnified oblique multislice (ZOOM) diffusion weighted imaging (DWI).

## Case presentation

**Fig. 1:**
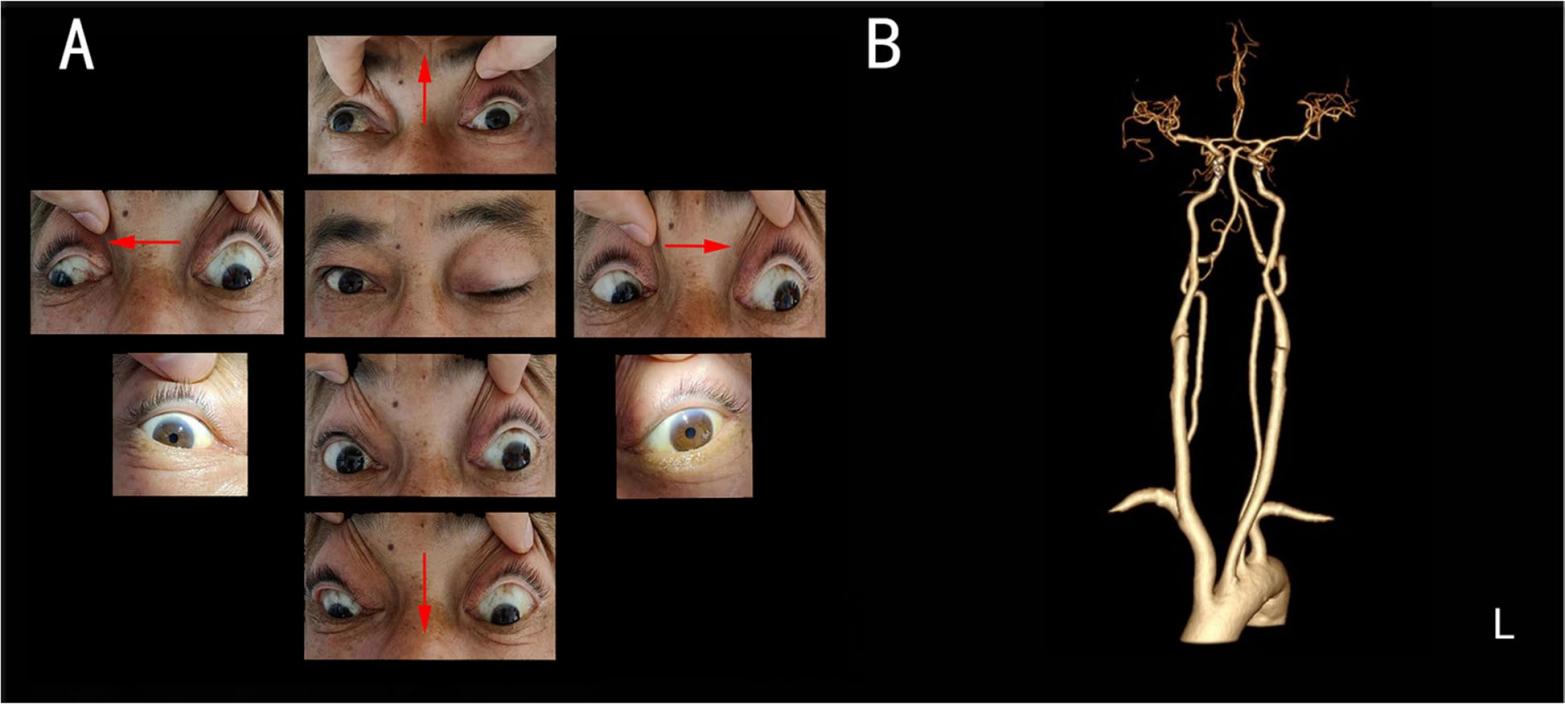
The pupillary sphincter is selectively spared in recorded eye movements (**A**); CT angiography of the brain vessels suggest the left vertebral artery originated directly from aortic arch (**B**). Abbreviations: L, left

**Fig. 2:**
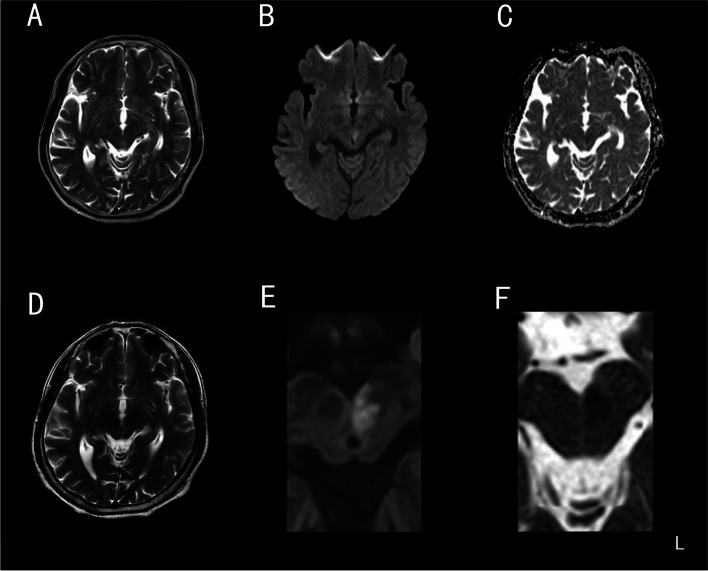
The mesencephalic periaqueductal slice T2WI (**A**), a suspicious restriction in conventional DWI (**B**), correlated apparent diffusion coefficient map (**C**), a corresponding T2WI of the ZOOM DWI (**D**), a verified restriction in the ZOOM DWI (**E**) and correlated apparent diffusion coefficient map (**F**). Abbreviations: L, left

A 55-year-old man, smoker, with hypertension and diabetes presented with a 3-h history of diplopia and drooping of his bilateral especially left eyelids without ocular discomfort or blurred vision. On neurological examination, there was an isolated oculomotor paresis consisting of left medial rectus, inferior oblique, superior rectus, inferior rectus with intact pupillary reflexes and bilateral especially left superior palpebral levator (Fig. 1A). Otherwise, the rest of the examinations including fatigability were unremarkable. Computed tomography (CT) angiography of the brain vessels showed left vertebral artery originated directly from the aortic arch, rather than the subclavian artery (Fig. 1B). Meanwhile, there were no other positive findings on CT images before and after injection of contrast agent. Conventional DWI with an in-plane resolution of 1.3 × 1.3 mm^2^ showed a suspicious restriction in the left midbrain periaqueductal region (Fig. 2) and verified by a specific technique, termed ZOOM DWI correlated with apparent diffusion coefficient map (Ingenia CX 3.0T, Philips). ZOOM DWI parameters were as follows: repetition time = 2500 ms, echo time = 85 ms, field of view = 97 × 39 mm^2^, matrix = 80 × 32, single shot echo plane imaging, slice thickness = 3 mm, b value = 1000 s/mm^2^, and 24 slices, providing with an in-plane resolution of 0.6 × 0.6 mm^2^ that can clearly distinguish the boundary between the target and the surrounding tissue [3, 4]. Thus, the patient was diagnosed with pure mesencephalic infarction. The recommended therapy was antiplatelet aggregation. The patient, therefore, received oral aspirin 100 mg/d, clopidogrel 75 mg/d and atorvastatin 20 mg/d. Gradual recovery of his palsy occurred within 3 months, and preventing cerebral infarction treatments were needed.

## Discussion

In clinical practice, we have often encountered a high signal intensity of the interpeduncle region of midbrain from neurologically healthy older adults on conventional axial DWI [5]. This need to be mentioned and evaluated, for identifying areas of abnormal diffusion optimally would be much more useful. As a small-field imaging technique reducing field of view, ZOOM DWI can identify restricted diffusion region without producing curl. Compared with conventional DWI images, ZOOM DWI has lower magnetic susceptibility artifact and higher image quality, and its advantages have been applied to the diagnosis of cervical spondylotic myelopathy [6]. Considering the caudal central subdivision of oculomotor nerve nuclei, located in the ventral periaqueductal region of midbrain, which is a single midline nucleus that supplies the superior palpebral levator on both sides, isolated nuclear oculomotor nerve palsy will produce bilateral ptosis [7]. The intra-axial oculomotor fascicular fibers are arranged from the lateral to medial side in the following order: the pupillary sphincter, inferior rectus, superior palpebral levator, medial rectus, superior rectus, and inferior oblique fibers. Due to the fascicular of left lateral side involved and medial most side spared, this case presented as ipsilateral dominant superior palpebral levator paralysis without pupil sphincter. The intra-axial oculomotor fascicular fibers are supplied by the inner and outer penetrating arteries of the superior medial mesencephalic branch (SMMB) of the posterior cerebral artery originated from the vertebrobasilar arteries, which are located medially and laterally respectively. The lateral occlusion of inner SMMB may speculate as the etiology, which blood flow supplies inferior oblique, superior rectus, medial rectus, superior palpebral levator, inferior rectus and pupillary sphincter from lateral to medial successively [8].

The left vertebral artery originated directly from the aortic arch but posterior cerebral artery lesions were not found in CT angiography. It is difficult to say whether it was the responsible factor for stroke or not, as one study has found association of posterior circulation stroke with vertebrobasilar system abnormalities [9]. Moreover, the patient had a history of diabetes, which usually presented as bilateral, incomplete oculomotor palsy [10] and less involved the superior palpebral levator. In addition, the mesencephalic infarction on ZOOM DWI could explain the symptoms and signs of the patient, so diabetic oculomotor palsy was not considered.

## Conclusion

The limited spatial resolution of conventional DWI is an enormous disadvantage if the clinical symptomatology indicates a lesion in the midbrain. For accurate diagnosis and evaluation of cerebral infarction, it is critical to identify signal alterations of mesencephalic interpeduncle area. ZOOM DWI technology provides higher accuracy for a small field of view than conventional does and should be recommended in selected patients presenting with specific midbrain syndromes. This is a rare presentation of partial oculomotor nerve palsy due to pure mesencephalic infarction especially verified by ZOOM DWI.

## Data Availability

No datasets were generated or analysed during the current study.

## Abbreviations

CT: Computed tomography
DWI: Diffusion weighted imaging
SMMB: Superior medial mesencephalic branch
ZOOM: Zonally magnified oblique multislice
